# Beyond One-Off Integrations: A Commercial, Substitutable, Reusable, Standards-Based, Electronic Health Record–Connected App

**DOI:** 10.2196/12902

**Published:** 2019-02-01

**Authors:** Kenneth D Mandl, Daniel Gottlieb, Alyssa Ellis

**Affiliations:** 1 Computational Health Informatics Program Boston Children's Hospital Boston, MA United States; 2 Department of Biomedical Informatics Harvard Medical School Boston, MA United States

**Keywords:** electronic medical records, application programming interfaces

## Abstract

The Substitutable Medical Apps and Reusable Technology (SMART) Health IT project launched in 2010 to facilitate the development of medical apps that are scalable and substitutable. SMART defines an open application programming interface (API) specification that enables apps to connect to electronic health record systems and data warehouses without custom integration efforts. The SMART-enabled version of the Meducation app, developed by Polyglot, has been implemented at scores of hospitals and clinics in the United States, nation-wide. After expanding their product’s reach by relying on a universal, open API for integrations, the team estimates that one project manager can handle up to 20 simultaneous implementations. The app is made available through the SMART App Gallery, an open app store that supports discovery of apps and, because the apps are substitutable, market competition. This case illustrates how a universal open API for patient and clinician-facing health IT systems supported and accelerated commercial success for a start-up company. Giving end users a wide and ever-growing choice of apps that leverage data generated by the health care system and patients at home through a universal, open API is a promising and generalizable approach for rapid diffusion of innovation across health systems.

## Introduction

The opportunity has never been greater to build a flourishing ecosystem of modern health care software [1]. Underpinnings of interoperability for data exchange and integration of diverse software have been developed, tested, and proven, technically and commercially. We present a health IT start-up’s trajectory as it transcended the common, frustrating modus operandi of *one-off* integrations of software for each new customer. Instead, the company leveraged an emerging, open, standards-based, national-scale, *app store* model. We believe that this success strongly supports an international strategy to deliver on health IT’s promises of evidence-based medicine, artificial intelligence, genomics, and value-based care, while promoting the success of innovators and empowering physicians and patients with the tools they need for 21st-century health care.

The Obama administration appropriated US $34 billion for incentivizing physicians and hospitals to purchase commercial electronic health records (EHRs) [2]. Because these products evolved from billing systems, clinical functionality was underdeveloped, noninteroperable, and not designed to exchange data [3]. A novel approach addressing these gaps, proposed in 2009 [4], was to connect apps to EHRs, much the same way mobile phone users select apps from app stores. Such apps would be substitutable: readily added to or deleted from the EHR across an application programming interface (API). APIs are fundamental to software produced by Apple, Google, Microsoft, Facebook, and Amazon and are used by billions of consumers. APIs enable software systems to interact with each other and exchange data. The Apple and Android APIs have spawned the creation of hundreds of thousands of apps without a need to devise subsequent interfaces, integrations, or agreements with Apple or Google.

To open EHRs to substitutable, third-party apps, the US Office of the National Coordinator of Health Information Technology funded the Substitutable Medical Apps and Reusable Technology (SMART) Health IT project [5-9]. The idea was that app developers, given an API specification that enabled access to the most commonly needed clinical data elements, including laboratory data, diagnoses, and medications, could readily create apps that could be added to or deleted from EHRs as easily as the (then 1-year-old) iPhone. Substitutability, a new approach to interoperability, was hoped to accelerate innovation by reducing barriers to integration and creating competition among developers; customers could select EHR-connected apps from an *app gallery*. Further, substitutability should enable customization of the end-user experience for providers using EHRs and patients accessing EHR portals.

In 2011, encouraged by the White House [10], the SMART team hosted a challenge to develop an app using the SMART API to connect to heath system data. A start-up company, Polyglot Systems, won for their app Meducation, designed to serve patients with low literacy levels, impaired vision, or language barriers. Meducation had been developed as a cloud-based medication management app, dynamically creating patient-specific medication instructions, and was designed to be intuitive and easy to read and understand in more than 20 languages. The user interface was designed through an iterative, user-centered design process with continual end-user feedback from IT teams at customer sites.

In under a week, the Polyglot team converted their existing app into a prototype SMART app that could be integrated with an EHR to pull the patient’s medication list across the API. The Polyglot team struggled a bit to understand how the clinical data (eg, medications and laboratories) were represented in the SMART API. In 2014, the SMART project [5] swapped out its own data models for the emerging international standard, Health Level Seven’s (HL7) Fast Health Interoperability Resources (FHIR) [11]. The contest also demonstrated the principle of reusability of apps, in that the Meducation app was able to readily run, without modification or customization, against three different clinical systems exhibiting the SMART API.

Polyglot pursued the use of the open SMART API in its strategy for integrating Meducation with EHRs and into patient and physician workflows. The company has since been acquired by First Databank, Inc. We explore implications of this approach for the rapidly evolving apps-driven health information economy [1].

## One-Off Commercial Integrations

SMART was not yet widely adopted when Polyglot won the contest. Therefore, their initial implementations required time-consuming and resource-intensive custom integrations for each clinical environment. Early commercial engagements were expensive for Polyglot and, since the company did not charge separately for implementation work, they incurred large costs. Further, the Polyglot team was limited to two to three implementations at any given time and required a third-party integrator to handle project management and resolve technical issues around integration.

One of Meducation’s initial implementations, prior to using SMART, was at a four-hospital system, HealthFirst, in Florida using the Allscripts Sunrise EHR. The project initiated in August 2015 and took 8 months to go live, after many integration challenges and higher-than-expected costs. There were issues in getting data to pass consistently from Allscripts Sunrise into the Meducation platform. Often the data were incomplete and missing information critical to drug safety. There were further issues in proper rendering of the user interface in the preferred workflow. System upgrades to Allscripts Sunrise often broke the interface and disrupted core functionalities, such as playing videos and rendering documents. Custom development projects came at a heavy price in time and effort, requiring substantial troubleshooting and testing.

These challenges were overcome through strong collaboration and a third-party integrator. The custom integration of a third-party app into a health system’s existing clinical systems is a huge undertaking. For this project, the hospital put together a project team of clinicians, pharmacists, and informatics personnel who met regularly to oversee the project and focus on end-user experience. The hospital’s IT group was brought on board early on to scope out the project and work with the Polyglot team on approaches to successful integration. The EHR vendor dedicated a team to the project for custom development work and an expert third-party integrator was brought in to deal with integration issues as they arose.

Some challenges, however, remained unresolved. For example, it would have been ideal for the Meducation document to be deposited into the EHR and included in the patient discharge instructions. This was not possible with the clinical systems and custom integration solutions available at the time, so end users needed to collate separate documents.

Subsequent implementations at clinical sites with an EHR system that Meducation had previously integrated with were more streamlined than the first-time implementation, generally taking only 3 months. The approach was to replicate the last working implementation with that EHR vendor and then troubleshoot for site-specific tailoring. Typical issues encountered during a second-time integration with a vendor include variations in the system versions, such as security settings and Internet browser type and version. Identifying and adapting to the differences across sites still required substantial involvement from the hospital’s IT group.

In contrast, starting a custom integration project at a clinical site with an EHR vendor with which Meducation had not yet implemented was like starting from scratch. The only lessons that could be carried over from other implementations were around the need for trustworthy and reliable partners, as well as the collaboration and communication that needs to be established for a successful integration. Not all implementation projects were successful. Reasons for failed integration were often related to the nature and complexity of the custom work.

## Advances in the SMART Ecosystem

The 21st Century Cures Act, signed into US law in 2016, requires that certified health IT products have an API that that allows consumers to access, exchange, and use their health information “without special effort” [12,13]. In the United States, most EHR vendors are using the *SMART on FHIR* API to satisfy this requirement [14-16]. EHR vendors have collaborated over the interpretation and profiling of the FHIR standard with selected terminologies (eg, RxNorm, Systematized Nomenclature of Medicine [SNOMED], and Logical Observation Identifiers Names and Codes [LOINC]) through the Argonaut Project [16]; they have incorporated support for SMART into their products and are beginning to roll out the technology to health care institutions. Epic and Cerner have SMART-enabled hundreds of their installs. Allscripts has SMART-enabled the releases of three of their products. Other EHR companies, including Athena and eClinical Works, have also built SMART support into their products.

Apple recently launched a new version of its Health app with a health-records feature for connecting with EHR instances, to enable a patient to download a copy of her records to her phone [17]. Hundreds of hospitals and health systems offer this capability.

## Technical and Business Efficiencies

Today, Meducation uses the SMART on FHIR API to access and integrate with clinical systems; a single project manager can oversee 20 or more highly standardized implementation projects without a third-party integrator. While early custom implementations of Meducation could not successfully integrate Meducation documents into the patient discharge instructions from the EHR, with SMART on FHIR, the company is increasingly able to accomplish this within the FHIR standard.

At Polyglot, SMART on FHIR has eliminated the need for large project teams and for third-party involvement. The team was able to configure their Meducation platform to the SMART on FHIR specifications by utilizing the SMART sandbox testing environment and sample patient datasets, which simulate a standards-compliant EHR.

When a health system expresses interest in Meducation, there is significantly less hesitation and resource allocation required from the hospital’s IT group and project management is much simpler. Integration of Meducation is standardized. There is little to no custom development needed to integrate the app with each vendor’s EHR system; thereby, testing and troubleshooting time is minimal.

For a new customer who is using an EHR system with which Meducation has not previously been integrated, they can nonetheless use a standard project plan, checklist, and resource list. None of the project management materials need to change based on the EHR system. Therefore, the project cost and timeline can be accurately estimated from the start. By relying on standard functionality regardless of the EHR system, the costs are much lower and much more predictable.

The standardization of the Meducation app on SMART has dramatically reduced the integration and implementation time by nearly eliminating the need for custom work; writing data back into EHRs still requires custom work in some cases, as the necessary components of the FHIR data model have not yet been universally implemented by vendors.

Though the app is still hosted through some legacy integrations with MEDITECH and Greenway Health, all other EHR implementations have migrated to substitutable patient-facing and provider-facing versions connecting via the SMART on FHIR API to Epic, Cerner Millennium, Allscripts Paragon, Allscripts Sunrise, eClinicalWorks, and athenahealth athenaClinicals. To date, the Meducation app has been implemented in over 3000 pharmacies, 60 clinics, and 70 hospitals in the United States nationwide. Of these, about 50 of the hospitals and 15 of the clinics are integrated via SMART.

**Figure 1:**
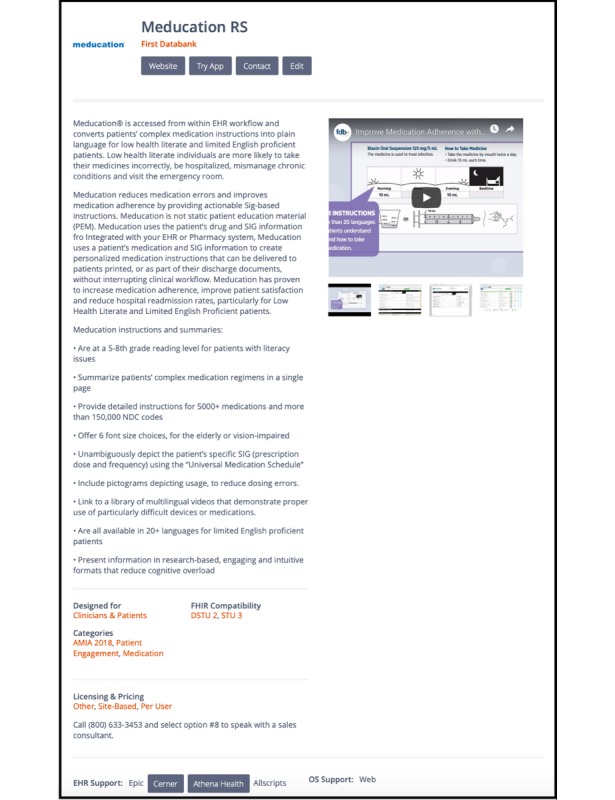
Meducation listing page on the SMART App Gallery [18].

**Figure 2:**
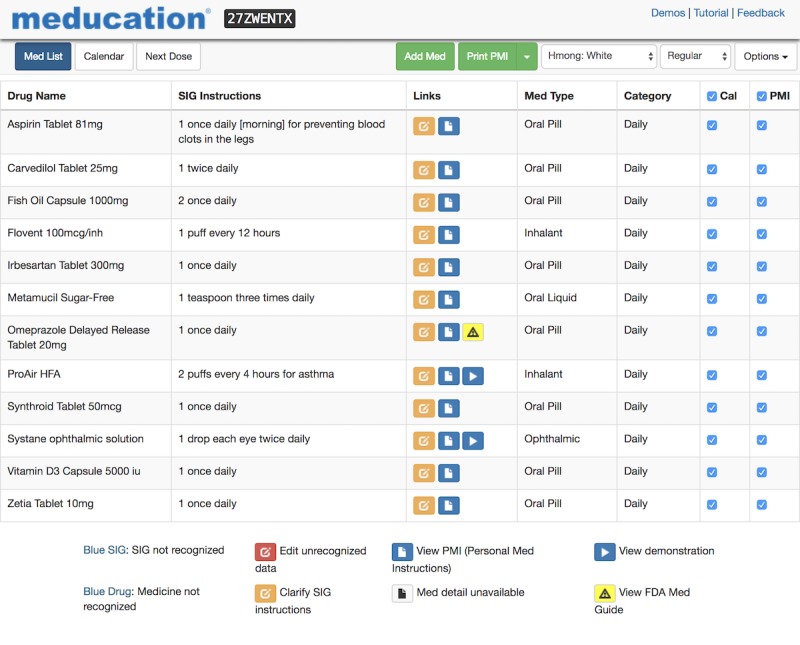
SMART sandbox demonstration data. Potential users can try the app by launching it against the demonstration data.

The current health apps marketplace includes the EHR vendor app stores, as well as iTunes and various Android app stores. The SMART App Gallery [18] is an open directory, showcasing SMART and FHIR apps with links to the various online stores where the app can be purchased. The Meducation app is listed in the SMART App Gallery (see Figure 1). The app utilizes the gallery’s demo functionality to enable users to launch the app against the sample clinical data in a sandbox environment, simulating the experience of using it in an EHR (see Figure 2). In addition, after terms were established with several EHR vendors, it has been listed in the Cerner App Gallery, Allscripts Application Store, and Athena Marketplace and will soon be listed in the Epic App Orchard.

As these emerging marketplaces for apps grow, the task of maintaining high-quality and safety levels for apps will be increasingly important. We expect this will occur through a mix of regulation [19], development-community standards [20], professional organization endorsements [21], third-party security certifications, and ongoing due diligence by health care organizations choosing to purchase and install the apps.

Innovative health app developers are using the SMART on FHIR open API specification to connect their products securely and efficiently into today’s health care ecosystem. Standardizing the integration process, as the Meducation team has done, eliminates the need for external, third-party services and enables efficiency and market scale for new innovations.

## Key Messages

APIs are ubiquitous in modern computing and enable software systems to interact with each other and exchange data. APIs have sparked ecosystems of apps for the iPhone and Android operating systems. They can similarly be used to drive an ecosystem of health care apps, essentially an *app store for health*.

In the United States, open and free API standards, developed, tested, and proven in the health care domain, have been integrated into EHR products and used to connect software to EHRs by both large (eg, Apple) and small software companies.

The experience of one commercial software company—Polyglot, recently acquired by First Databank, Inc—cogently illustrates how an open, consistent, standards-based API can foster innovation as well as commercial success.

## Abbreviations

API: application programming interface
EHR: electronic health record
FHIR: Fast Health Interoperability Resources
HL7: Health Level Seven
LOINC: Logical Observation Identifiers Names and Codes
SMART: Substitutable Medical Apps and Reusable Technology
SNOMED: Systematized Nomenclature of Medicine
